# Pre-exercise Caffeine Intake Enhances Bench Press Strength Training Adaptations

**DOI:** 10.3389/fnut.2021.622564

**Published:** 2021-01-26

**Authors:** Verónica Giráldez-Costas, Carlos Ruíz-Moreno, Jaime González-García, Beatriz Lara, Juan Del Coso, Juan José Salinero

**Affiliations:** ^1^Exercise Physiology Laboratory, Camilo José Cela University, Madrid, Spain; ^2^Centre for Sport Studies, Rey Juan Carlos University, Fuenlabrada, Spain; ^3^Faculty of Sport Sciences, Castilla-La Mancha University, Toledo, Spain

**Keywords:** resistance exercise, muscle strength, resistance training, stimulant, ergogenic aid

## Abstract

Previous research has identified acute caffeine intake as an effective ergogenic aid to enhance velocity and power during bench press exercise. However, no previous investigation has analyzed the effects of chronic intake of caffeine on training adaptations induced by bench press strength training. Thus, the aim of this investigation was to determine the effects of pre-exercise caffeine intake on training adaptations induced by a bench press training protocol. Using a double-blind, randomized experimental design, 16 healthy participants underwent a bench press training protocol for 4 weeks (12 sessions). Seven participants ingested a placebo and nine participants ingested 3 mg/kg/BM of caffeine before each training session. Three days before, and 3 days after the completion of the training protocol, participants performed a one-repetition maximum (1RM) bench press and force-velocity test (from 10 to 100% 1RM). From comparable pre-training values, the strength training similarly increased 1RM in the caffeine and placebo groups (+13.5 ± 7.8% vs. +11.3 ± 5.3%, respectively; *p* = 0.53). In the caffeine group, the strength training induced a higher mean velocity at 40%, (0.81 ± 0.08 vs. 0.90 ± 0.14 m/s), 60% (0.60 ± 0.06 vs. 0.65 ± 0.06 m/s), 70% (0.47 ± 0.05 vs. 0.55 ± 0.06 m/s), 80% (0.37 ± 0.06 vs. 0.45 ± 0.05 m/s), 90% (0.26 ± 0.07 vs. 0.34 ± 0.06 m/s), and 100% 1RM (0.14 ± 0.04 vs. 0.25 ± 0.05 m/s; *p* < 0.05) while the increases in the placebo group were evident only at 30 (0.95 ± 0.06 vs. 1.03 ± 0.07 m/s), 70% (0.51 ± 0.03 vs. 0.57 ± 0.05 m/s) and 80% 1RM (0.37 ± 0.06 vs. 0.45 ± 0.05 m/s) (*p* < 0.05). The placebo group only increased peak velocity at 60 and 70% 1RM (*p* < 0.05) while peak velocity increased at 10%, and from 30 to 100% 1RM in the caffeine group (*p* < 0.05). The use of 3 mg/kg/BM of caffeine before exercise did not modify improvements in 1RM obtained during a 4 week bench press strength training program but induced more muscle performance adaptations over a wider range of load.

## Introduction

Caffeine (1,3,7 trimethylxanthine) is one of the most commonly consumed substances in the world, despite a lack of any nutritional value (1). Within a sport setting, caffeine supplementation is a popular strategy used to improve physical performance (2, 3). Approximately, 76% of elite athletes' post-competition urine contains caffeine and this substance is present in the post-competition urine of athletes of most sport specialties (4). The ubiquitous presence of caffeine in these urine samples suggests that athletes of very differing sport disciplines use caffeine supplementation before or during competition. Use of caffeine in sport is supported by recent evidence that confirms the ergogenic properties of acute caffeine intake (from 3 to 9 mg of caffeine per kilogram (kg) of body mass; mg/kg/BM) in a number of exercise modalities (5–10).

The main mechanism behind caffeine ergogenicity is its capacity to blunt the fatiguing effects of adenosine (11). After ingestion and distribution through several tissues, including crossing the blood brain barrier, caffeine blocks specific adenosine receptors (12) preventing the inhibition that adenosine causes on the central nervous system (CNS) (13). Therefore, acute caffeine intake produces a stimulation of the CNS that facilitates the recruitment of muscle fibers during maximal and submaximal muscle contractions (14). As adenosine is a subproduct of different metabolic pathways, the effect of caffeine on the CNS successfully enhances physical performance in a myriad of exercise activities, but it may be especially effective to enhance muscle performance. Evidence in resistance-based exercise indicates ergogenic benefits of acute caffeine intake during bench press maximal strength, muscle power output and muscular strength-endurance (15–19), with recent meta-data confirming a moderate-to-high effect of caffeine on resistance-based exercise (20). In addition, pre-exercise caffeine intake has the capacity to increase the total amount of work performed during a strength training session due to more power being produced in each repetition (16) or due to more repetitions executed with caffeine during the whole session (21).

These outcomes suggest that caffeine is useful to enhance several forms of resistance-based exercise. However, there is a lack of research on the chronic effects of caffeine intake along with a resistance training program to ascertain if the acute effects of caffeine during resistance exercise may help to augment the improvements in velocity and power output induced by strength training when caffeine is ingested before each training session. Hence, the aim of this investigation was to determine the effect of pre-exercise caffeine intake on training adaptations induced by a bench press training protocol of 4 weeks (12 training sessions). Our research group hypothesized that individuals who ingested caffeine before exercise during the strength training program would obtain greater adaptations over those who were supplemented with a placebo.

## Methods

### Participants

Sixteen healthy participants (12 men and 4 women) volunteered to participate in this investigation. They presented a mean ± standard deviation age of 27.9 ± 7.2 years; body mass of 71.7 ± 10.0 kg; body height of 173.0 ± 7.0 cm; body fat percentage of 18.3 ± 8.1%; and bench press one-repetition maximum of 60.4 ± 17.8 kg. All of the participants fulfilled the following inclusion criteria: (a) age between 18 and 45 years old; (b) caffeine naïve or low habitual caffeine consumers (<0.99 mg/kg/BM/day), as previously suggested by Filip et al. (22); and (c) more than 1 year of resistance training experience. Participants were excluded if they reported (a) upper body injury within the previous 6 months; (b) a positive smoking status; (c) medication usage within the previous month; (d) a previous history of cardiopulmonary diseases; (e) allergy to caffeine; or (f) use of oral contraceptive pills, as they may interfere with caffeine pharmacokinetics (23). Participants were encouraged to maintain a diet following previous nutritional guidelines to assure carbohydrate (24) and protein availability (25), and correct hydration during the whole experiment (26) and to distribute foods in five meals throughout the day. Participants wrote their diets down in a personal journal and a subsequent analysis ensured that all participants had >2,600 kcal/day in the men and >2,200 kcal/day in the women, >5 g/kg/BM/day of carbohydrate, and >1.6 g/kg/BM/day of protein (PCN 1.0 software, Cesnid, Spain). Participants were encouraged to maintain their aerobic exercise habits (i.e., running, cycling, etc.) throughout the duration of the study to avoid any detraining effects and to refrain from vigorous exercise for at least 48 h prior to testing. They were advised to avoid any form of upper-body strength or upper-body and lower-body resistance exercise during the duration of the investigation, to avoid the interference of other resistance exercise activities on the results of the investigation. Lastly, participants were asked to abstain from any form of dietary caffeine intake and from dietary supplement use for the duration of the study. They provided their informed consent prior to participating in the investigation after having been informed of the experimental procedures and risks. The study was approved by the Camilo José Cela University Research Ethics Committee and was conducted in accordance with the latest version of the Declaration of Helsinki.

### Experimental Design

**Figure 1:**
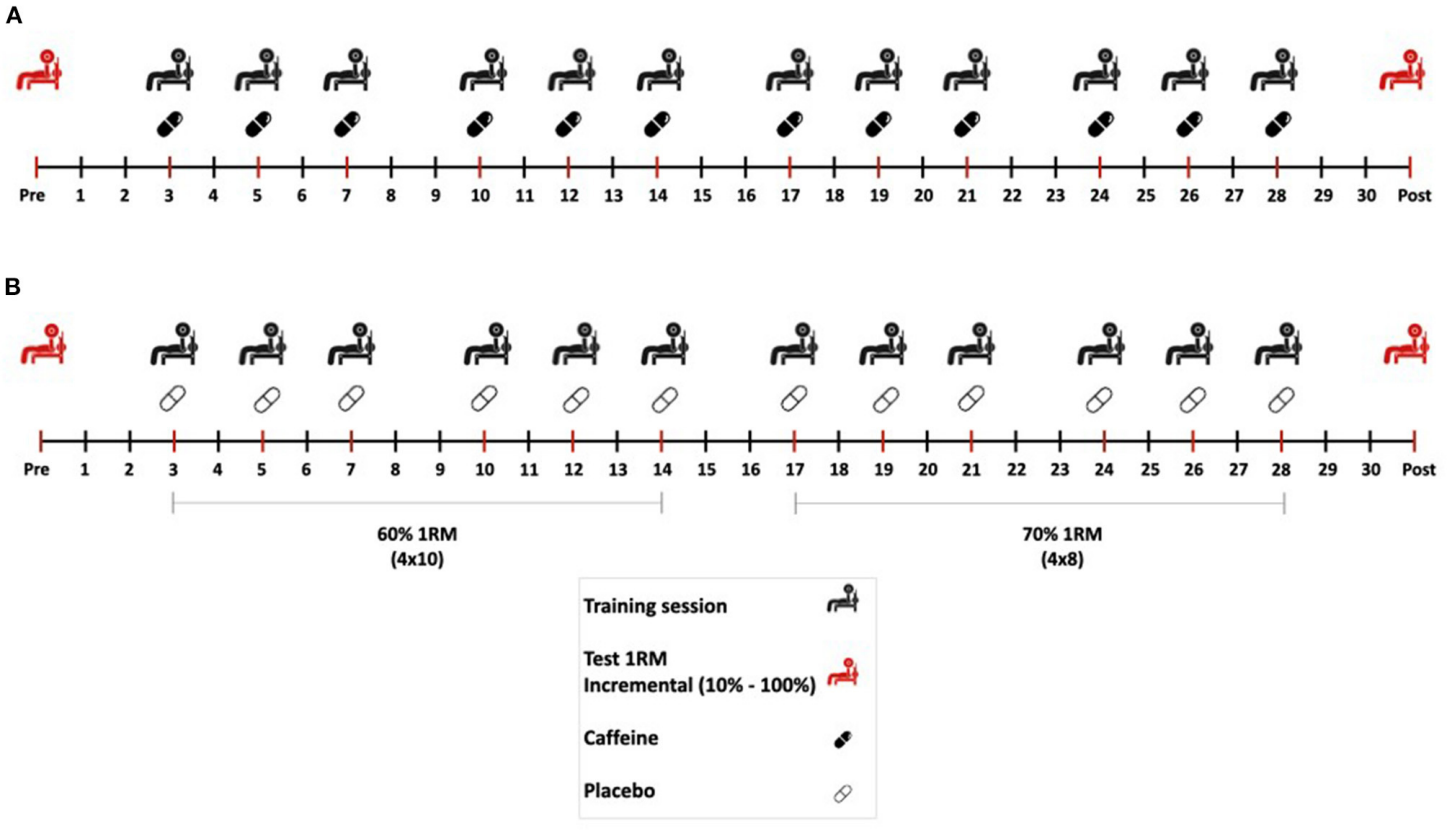
Experimental design of the investigation. Participants underwent a strength training protocol consisting of 12 training sessions in the bench press exercise for 4 weeks. During the first 2 weeks, participants performed 4 sets of 10 repetitions at 60% 1RM and in the last 2 weeks they performed 4 sets of 8 repetitions at 70% 1RM. Before each training session, nine participants ingested a capsule containing 3 mg/kg/BM of caffeine **(A)** and seven participants ingested a capsule containing a placebo **(B)**. Just before and after the strength training protocol, participants performed a one-repetition maximum test in the bench press exercise and a force-velocity test using incremental loads.

This investigation followed a longitudinal, double-blind, randomized experimental design. All participants performed a 4 week strength training protocol consisting of 12 training sessions in the bench press exercise, with a frequency of 3 sessions per week. There were at least 48 h between sessions, and they were performed on Monday, Wednesday and Friday of each week. All participants performed the training sessions in the morning between 9:00 and 12:00 a.m. and at least 3 h after breakfast. They were randomly allocated to a caffeine group (*n* = 9) or to a placebo group (*n* = 7). Both groups were instructed to ingest an opaque and unidentifiable capsule 1 h before the onset of each training session. The capsule was prepared by a specialized researcher blinded to the experiment outcomes and the ingestion was confirmed by another researcher. In the caffeine group, the capsule contained caffeine (3 mg/kg/BM; 100% purity, Bulk Powders, UK) while in the placebo group the capsule contained an inert substance (cellulose; 100% purity, Guinama, Spain). Just before and after the strength training protocol, participants performed a one-repetition maximum test (1RM) in the bench press exercise and a force-velocity test using loads from 10 to 100% 1RM, as measured in the previous test. Of note, participants did not ingest any capsule before the pre-training and post-training sessions to isolate the effect of caffeine on the strength training protocol. The trials and training sessions were performed in a laboratory setting with controlled ambient temperature (~21°C). Figure 1 displays the study design.

### Testing Procedures

On the day of the pre-training experimental trial, participants arrived at the laboratory in the morning (between 9.00 and 12.00 a.m.) in a fed state (~3 h after their last meal). Upon arrival, they were weighed unclothed (±50 g, Radwag, Poland) in order to properly calculate caffeine dosage for the experiment. Body fat percentage was subsequently estimated by bioelectrical impedance (model BC-418, Tanita, Japan). For 1RM measurements in the bench press exercise, participants performed a 15 min standardized warm-up consisting of upper-body joint mobility exercises followed by sets of increasing loads estimated to be between 20 and 90% of the participant's 1RM (27). Then, bench press 1RM was measured with a maximum of five maximal attempts permitted and 5 min of recovery between attempts. The 1RM was identified as the maximum successful lift with a correct technique and it was used to standardize the load in the following force-velocity test and subsequent training sessions. Twenty minutes after 1RM measurement, the force-velocity test was performed with loads between 10% and 100 1RM, using 10% increments. Participants performed two maximal repetitions of the bench press exercise, with the purpose of obtaining peak bar velocity during the concentric phase of the movement with each load, and the best repetition was selected for analysis. If participants considered that the repetition was not maximal, they were allowed to repeat any attempt. Standardized verbal encouragement was used for all loads to aid participants to obtain the highest velocity (28) and they received velocity performance feedback immediately after each repetition. Execution technique was monitored by two experienced researchers for reliability of the experimental conditions. The testing was performed on a Smith Machine (Technogym, Barcelona, Spain) in which two vertical guides regulated the barbell movement. In each attempt, barbell velocity in the concentric phase of the movement was recorded with a rotatory encoder and its associated software (1,000 Hz, Isocontrol, EV-Pro, Spain) and mean and peak velocity (m/s) were measured. Mean and peak power (W) were calculated using the load in kg. The pre-training test was performed 72 h before the first training session and the post-training test was performed 72 h after the last training session.

### Training Program

The training program lasted 4 weeks and included twelve training sessions in the bench press exercise. The exercise was performed in the same Smith machine employed for the testing. During the first 2 weeks, participants performed 4 sets of 10 repetitions at 60% of the pre-training 1RM and in the last 2 weeks the training protocol was changed to 4 sets of 8 repetitions at 70% of the pre-training 1RM. The training program was designed with the aim of maximizing increases in maximum/peak velocity/power in intermediate loads. Before the onset of the training protocol, participants received a plastic container with 12 unidentifiable capsules which were filled with caffeine or placebo according to the group to which they were allocated. Before each training session, participants ingested the assigned capsule 60 min before the onset of exercise and rested supine for 1 h. Afterwards, they carried out a standardized 15 min warm-up including upper body exercises and 3 sets of 10 ballistic repetitions of the bench press throwing with a load that represented 30–40% of their 1 RM. After warming up, participants performed the training set for the session. The movement tempo during the exercise was 1/2/X/1 (one second for the eccentric phase, a 2 s pause during the transition phase from the eccentric to the concentric phase, with X referring to the highest possible velocity during the concentric phase of movement, and the last digit indicating a 1 s pause between the concentric and eccentric phases (16, 29). There was a 3 min recovery period between sets. The participants were encouraged to perform their lifts at peak velocity during each bench press repetition. Standardized verbal encouragement was used for all participants (28) and they received visual feedback between sets and at the end of the training session on power and velocity results. Execution technique was monitored by two experienced researchers, blinded to the treatment assigned to each participant, for reliability of the experimental conditions.

### Statistical Analysis

Statistical analyses were carried out using the software IBM SPSS Statistics for Macintosh, Version 26.0 (IBM Corp., Armonk, NY, US). Data were normally distributed in all variables as determined by the Shapiro-Wilk test. Additionally, the sphericity assumption was checked with Mauchly's test. If this assumption presented a probability of *p* < 0.05, the Greenhouse-Geisser correction was used. At each load, a two-way (substance × time) within-between participants analysis of variance (ANOVA) was used to identify the effects of caffeine and of the training intervention. Additionally, effect sizes (ES) were calculated between pairs using Cohen's *d* (±95% confidence intervals) and they were interpreted according to the following thresholds: <0.2 trivial, >0.2–0.6 small, >0.6–1.2 moderate, >1.2–2.0 large, and >2 very large. Results are expressed as mean ± standard deviation. The significance level was set at *p* < 0.05.

## Results

### One Repetition Maximum

**Figure 2:**
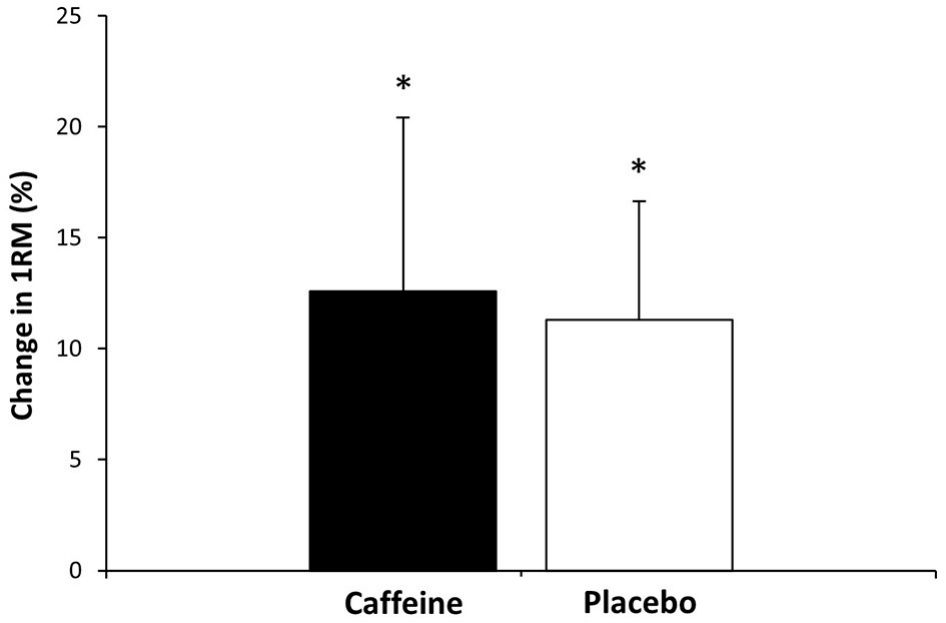
Change in one-repetition maximum (1RM) in the bench press exercise after a 4 week resistance training protocol (12 training sessions) with the ingestion of 3 mg/kg/BM of caffeine (*n* = 9) or a placebo (*n* = 7) prior to each training session. Data are shown as mean ± SD. (^*^) Difference between pre-training and post-training within the same group at *p* < 0.05.

The change in 1RM induced by the training protocol was similar in both groups [*p* = 0.53; *F*_(1, 14)_ = 0.42, Figure 2]. Moreover, from similar pre-training values (62.50 ± 19.02 and 58± 15.98 kg, for caffeine and placebo, respectively; *p* = 0.11; ES = 0.06), the resistance training increased 1RM values in both the caffeine (70.13 ± 20.08 kg, *p* < 0.01; ES = 0.30) and placebo groups (64.19 ± 17.12 kg, *p* < 0.01; ES = 0.30).

### Mean Velocity

**Figure 3:**
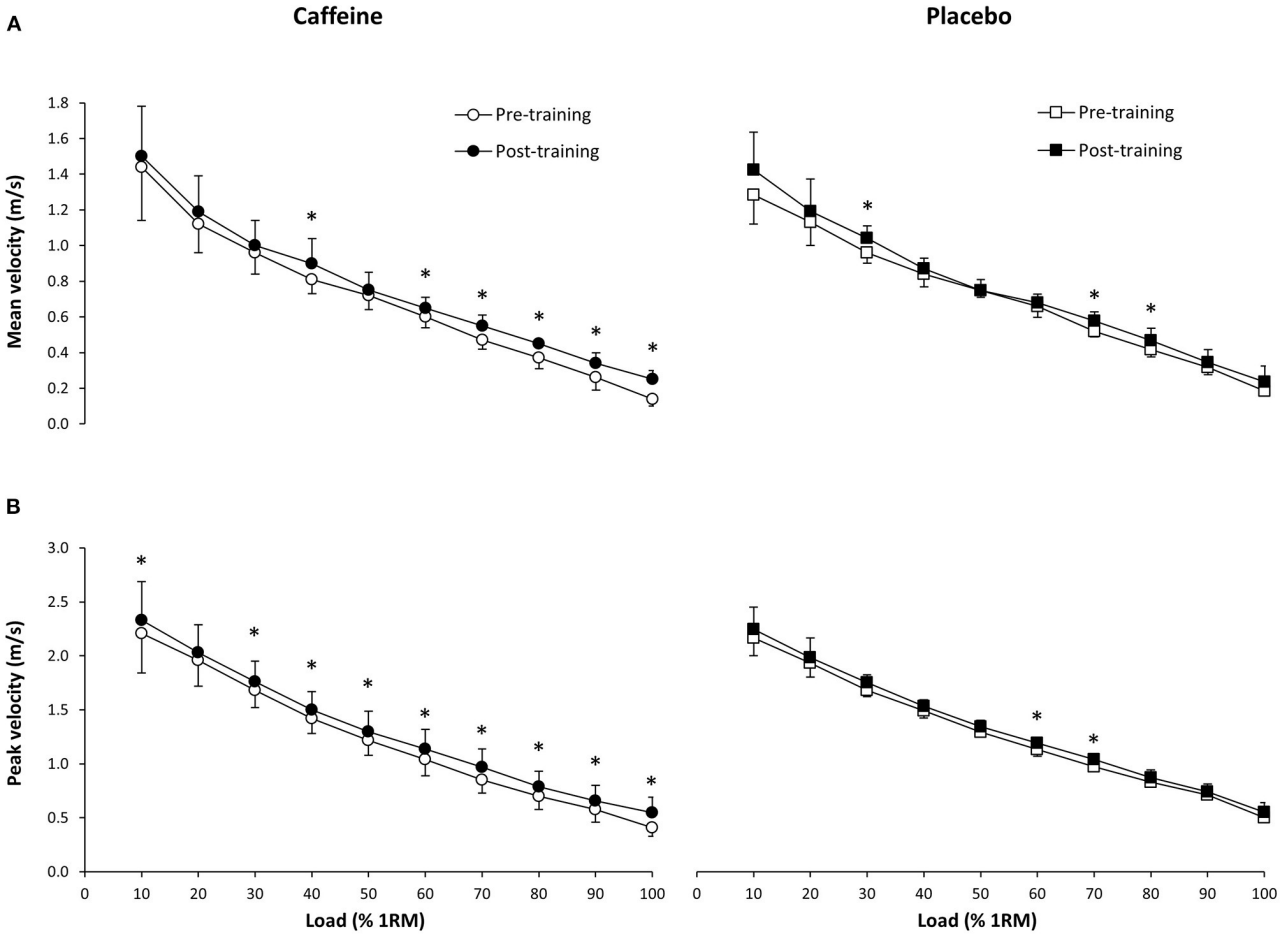
Mean velocity **(A)** and peak velocity **(B)** during a bench press exercise at different loads (from 10 to 100% of one repetition maximum; 1RM) before and after 4 weeks of resistance training (12 training sessions) with the ingestion of 3 mg/kg/BM of caffeine (*n* = 9) or a placebo (*n* = 7) prior to each training session. Data are shown as mean ± SD for each load. (^*^) Difference between pre-training and post-training within the same group at *p* < 0.05.

There were no differences in mean velocity changes between groups at any load (*p* > 0.05). Nevertheless, in the caffeine group, there was an increase in mean velocity at 40% (0.81 ± 0.08 vs. 0.90 ± 0.14 m/s; *p* = 0.01, ES = 0.91), 60% (0.60 ± 0.06 vs. 0.65 ± 0.06 m/s; *p* = 0.02, ES = 0.74), 70% (0.47 ± 0.05 vs. 0.55 ± 0.06 m/s; *p* < 0.01, ES = 1.50), 80% (0.37 ± 0.06 vs. 0.45 ± 0.05 m/s; *p* < 0.01, ES = 1.00), 90% (0.26 ± 0.07 vs. 0.34 ± 0.06 m/s; *p* < 0.01, ES = 0.76), and 100% 1RM (0.14 ± 0.04 vs. 0.25 ± 0.05 m/s; *p* < 0.01; ES = 2.07; Figure 3A). In the placebo group, mean velocity only increased at 30% (0.95 ± 0.06 vs. 1.03 ± 0.07 m/s; *p* = 0.02, ES = 1.07), 70% (0.51 ± 0.03 vs. 0.57 ± 0.05 m/s; *p* = 0.01, ES = 1.27), and 80% 1RM (0.37 ± 0.06 vs. 0.45 ± 0.05 m/s *p* = 0.02, ES = 0.90).

### Peak Velocity

There were no differences in peak velocity changes between groups at any load (*p* > 0.05). However, in the caffeine group, the training program increased peak velocity at 10% (2.21 ± 0.37 vs. 2.33 ± 0.36 m/s; *p* = 0.01, ES = 0.26), 30% (1.68 ± 0.16 vs. 1.76 ± 0.19 m/s; *p* = 0.04, ES = 0.43), 40% (1.42 ± 0.14 vs. 1.50 ± 0.17 m/s; *p* = 0.01, ES = 0.48), 50% (1.22 ± 0.14 vs. 1.30 ± 0.19 m/s; *p* = 0.02, ES = 0.24), 60% (1.04 ± 0.15 vs. 1.14 ± 0.18 m/s; *p* < 0.01, ES = 0.55), 70% (0.85 ± 0.12 vs. 0.97 ± 0.17 m/s; p <0.01, ES = 0.74), 80% (0.70 ± 0.12 vs. 0.79 ± 0.14 m/s; *p* = 0.02, ES = 0.56), 90% (0.58 ± 0.12 vs. 0.66 ± 0.14 m/s; *p* = 0.04, ES = 0.44), and 100% 1RM (0.41 ± 0.08 vs. 0.55 ± 0.14 m/s; *p* < 0.01, ES = 1.11; Figure 3B). In the placebo group, peak velocity only increased at 60% (1.13 ± 0.07 vs. 1.19 ± 0.06 m/s; *p* = 0.03, ES = 0.84) and 70% 1RM (0.97 ± 0.07 vs. 1.04 ± 0.06 m/s; *p* = 0.03, ES = 0.82).

### Mean Power

**Figure 4:**
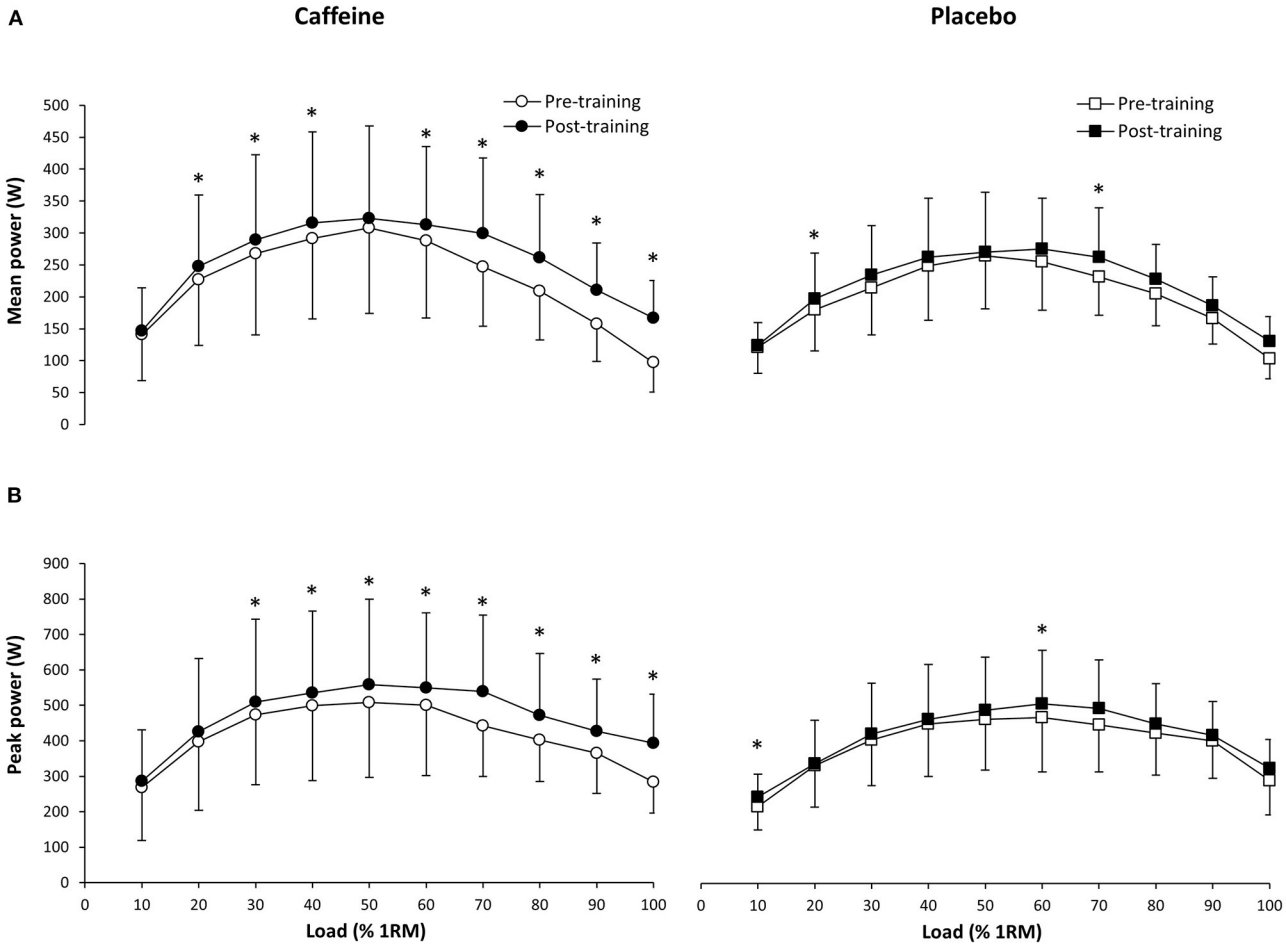
Mean power **(A)** and peak power **(B)** during a bench press exercise at different loads (from 10 to 100% of one repetition maximum; 1RM) before and after 4 weeks of resistance training (12 training sessions) with the ingestion of 3 mg/kg/BM of caffeine (*n* = 9) or a placebo (*n* = 7) prior to each training session. Data are shown as mean ± SD for each load. (^*^) Difference between pre-training and post-training within the same group at *p* < 0.05.

There were no differences in mean changes between groups at any load (*p* > 0.05). In the caffeine group, the training program increased mean power output at 20% (227.06 ± 103.09 vs. 247.68 ± 111.71 W; *p* = 0.01, ES = 0.14), 30% (268.22 ± 127.56 vs. 288.97 ± 133.55 W; *p* = 0.03, ES = 0.12), 40% (291.24 ± 125.56 vs. 315.85 ± 142.53 W; *p* < 0.05, ES = 0.11), 60% (287.74 ± 121.26 vs. 313.06 ± 122.15 W; *p* = 0.01, ES = 0.18), 70% (246.79 ± 93.09 vs. 299.39 ± 117.98 W; *p* < 0.01, ES = 0.39), 80% (209.35 ± 76.75 vs. 261.62 ± 98.73 W; *p* < 0.01, ES = 0.48), 90% (157.61 ± 58.56 vs. 210.40 ± 74.01 W; *p* < 0.01, ES = 0.66), and 100% 1RM (96.77 ± 45.62 vs. 166.90 ± 58.84 W; *p* < 0.01, ES = 1.10; Figure 4A). In the placebo group, mean power output only increased at 20% (179.71 ± 64.16 vs. 197.16 ± 71.03 W; *p* = 0.04, ES = 0.18) and 70% 1RM (230.99 ± 59.61 vs. 261.96 ± 76.47 W; *p* = 0.03, ES = 0.34).

### Peak Power

There were no differences in peak power changes between groups at any load (*p* > 0.05). In the caffeine group, the training program increased peak power output at 30% (473.14 ± 197.49 vs. 509.18 ± 233.10 W; *p* = 0.02, ES = 0.10), 40% (499.84 ± 212.52 vs. 535.68 ± 230.48 W; *p* = 0.02, ES = 0.10), 50% (508.01 ± 210.92 vs. 558.51 ± 241.32 W; *p* = 0.02, ES = 0.17), 60% (501.03 ± 199.59 vs. 550.08 ± 211.20 W; *p* < 0.01, ES = 0.19), 70% (442.13 ± 142.34 vs. 538.95 ± 215.49 W; *p* < 0.01, ES = 0.39), 80% (402.08 ± 116.70 vs. 471.99 ± 174.43 W; *p* = 0.03, ES = 0.34), 90% (365.14 ± 113.43 vs. 427.31 ± 146.91 W; *p* < 0.05, ES = 0.37), and 100% 1RM (283.43 ± 87.18 vs. 393.35 ± 138.39 W; *p* < 0.01, ES = 0.80; Figure 4B). In the placebo group, the training protocol only induced a difference in peak power output at 10% (215.51 ± 65.88 vs. 242.22 ± 63.91 W; *p* = 0.03, ES = 0.32) and 60% 1RM (465.57 ± 153.15 vs. 503.33 ± 149.75 W; *p* = 0.04, ES = 0.20).

## Discussion

The aim of this study was to investigate the effect of pre-exercise caffeine intake on muscle performance adaptations obtained by a bench press strength training protocol lasting 4 weeks. This research question was designed to determine whether caffeine can be effectively used to improve bench press performance as a chronic supplementation routine for training, as previous studies in this field have reported acute benefits from the ingestion of a single dose of caffeine (16, 18, 21). To the authors' knowledge, this is the first research to analyze the concomitant effects of strength training and chronic caffeine intake on bench press performance. The main outcomes of this investigation indicate that, in comparison to a placebo, the intake of 3 mg/kg/BM of caffeine 1 h before the onset of the workout for 12 sessions increased the number of training-induced adaptations in mean and peak values for both velocity and power over the placebo (Figures 3, 4). Specifically, the magnitude of the change on the velocity-load and power-load curves was higher with caffeine than with a placebo while caffeine induced these improvements over a wider range of loads. Chronic caffeine intake did not enhance the pre-to-post-training change in the weight lifted during the 1RM test. Therefore, our results suggest that the pre-exercise use of caffeine associated with a strength training program may be useful to enhance the adaptations in muscle performance at submaximal loads in the bench press exercise.

Previous research has shown that acute caffeine intake induces several improvements in variables associated with bench press performance (16, 21, 30, 31). In the light of these outcomes, there is a consensus to consider acute caffeine intake as an effective supplementation protocol to improve performance in the bench press exercise. Based upon its acute effects, it has been suggested that caffeine may be an effective ergogenic aid when used chronically as a pre-exercise supplementation, to enhance the adaptations induced by a strength training program (3). However, no previous investigation has determined the chronic effects of caffeine intake along with a resistance training program to confirm this hypothesis. The current investigation confirms this suggestion as the intake of 3 mg of caffeine per kg of body mass before each of the 12 sessions that composed the training program was effective to increase the improvements of the program on bar velocity and power developed during the bench press exercise. The use of caffeine was effective to induce a higher number of statistically significant pre-to-post training differences for both velocity and power over a wider range of loads, without modifying the change induced in 1RM over the placebo. This is a novel finding in the literature that points toward the efficacy of using pre-exercise caffeine supplementation to enhance training adaptations, at least for resistance-based exercise. However, the training program only lasted 4 weeks and further investigations are needed with longer training programs, with different exercises, with different doses of caffeine and with different timings for caffeine intake, to reveal if caffeine can exert ergogenic effects in other resistance exercise programs, particularly due to the potential tolerance to caffeine ergogenicity induced by chronic ingestion (32).

As load influences the movement velocity and the power output in the bench press exercise (33) it is necessary to consider the possible ergogenic effects of caffeine along the whole velocity-load and the power-load spectrum. Previous research (31) showed that caffeine increases the mean concentric velocity in the bench press at high loads (90% 1RM) more than at lower loads (50 or 75% 1RM). In our study, caffeine induced changes in mean velocity at 40% 1RM, and in all loads from 60 to 100% 1RM (Figure 3A). These changes may be associated with the training protocol used in this investigation as participants trained at 60% 1RM during the first 2 weeks and at 70% 1RM during the last 2 weeks, always using peak velocity in each repetition. Interestingly, caffeine did not affect the change in 1RM induced by the training, likely due to the lack of maximal loads used during the training program. Although it was not investigated here, it is likely that caffeine would have enhanced 1RM more than the placebo in the case of using a training program specifically designed to produce improvements in maximal strength (i.e., with some sessions including loads at 90–100% 1RM). To date, this is speculation that requires confirmation by future research. In any case, our findings suggest that training with pre-exercise caffeine supplementation may increase mean bar velocity (and therefore, mean power output) in a wider range of loads than the same training protocol without caffeine supplementation. Further investigations combining caffeine and a variety of training weights are necessary to determine the outcome of pre-exercise caffeine intake in the whole spectrum of loads.

The experimental design employed in this investigation presents some limitations that should be addressed to enhance the application of the results. First, the training program consisted of 4 sets of 8–10 bench press executions at 60–70% 1RM performed at peak velocity in a group of individuals with moderate resistance training experience and with a certain heterogeneity in 1RM values. However, it is necessary to determine if caffeine may also enhance muscle performance when using other resistance-training scenarios (i.e., with different numbers of sets, repetitions, and loads), with longer training programs, and with different doses and times of caffeine administration. As the study sample was composed of individuals with moderate resistance exercise experience, the translation of the research outcomes to highly trained athletes should be made with caution. Second, this investigation used two groups of individuals randomly allocated to the caffeine and placebo groups. Still, it is possible that part of the differences in the response to training may be associated with interindividual differences in the adaptation to strength training (34). The use of cross-over experimental designs is needed to confirm the benefits of chronic pre-exercise caffeine intake for resistance exercise programs. Third, this study did not include blood and tissue samples and thus we have no data about serum caffeine concentrations in the individuals in the caffeine group nor the mechanism that induced a higher training response with caffeine. The assessment of serum caffeine concentration could have been useful to determine whether participants performed the training sessions with peak concentrations. In addition, the assessment of how many participants correctly guessed the group they had been allocated to would also have improved the quality of this experiment. Fourth, diet during the training program was recorded and controlled to ensure all participants consumed adequate amounts of energy, carbohydrate, and protein, but they were not standardized. The control of diet, particularly in the hours after the end of each workout, may have limited the interference of post-training feeding on the results of this investigation. Lastly, we used a group of individuals with low habituation to caffeine. As there is tolerance to the caffeine ergogenic effect in endurance and anaerobic-like exercise (32), it is probable that the effect found in this investigation would be smaller in individuals habituated to this substance. Therefore, it could be hypothesized that higher doses of caffeine may be necessary for individuals habituated to caffeine (19, 35) or when caffeine is used for periods longer than 4 weeks.

In summary, pre-exercise ingestion of 3 mg/kg/BM of caffeine did not modify improvements in 1RM obtained during a 4 week bench press strength training program. However, chronic pre-exercise caffeine ingestion increased the number of training-induced adaptations in mean and peak values for both velocity and power over the placebo. Caffeine moved the velocity-load and the power-load curves upwards to a greater extent with caffeine than with the placebo, with statistically significant differences over a wider range of loads. From a practical point of view, caffeine seems to be an effective ergogenic aid for upper-body strength training at moderate loads when ingested before exercise. Sport science practitioners and nutritionists could incorporate the use of caffeine in their interventions with athletic cohorts, but keeping in mind the drawbacks of caffeine supplementation such as the progressive importance of some side effects when the substance is consumed chronically (36). Elite athletes and recreational lifters might consider the use of caffeine for strength training, given the benefits of this substance, although the use of caffeine should be controlled by a professional to avoid excessively high doses and habituation.

## Data Availability Statement

The original contributions presented in the study are included in the article/supplementary material, further inquiries can be directed to the corresponding author/s.

## Ethics Statement

The studies involving human participants were reviewed and approved by Institutional Review Board of the Camilo José Cela University. The patients/participants provided their written informed consent to participate in this study.

## Author Contributions

VG-C, CR-M, JG-G, BL, JD, and JS conceived and designed the investigation. VG-C, CR-M, JG-G, and JS collected the data. VG-C, BL, JD, and JS analyzed and interpreted the data. VG-C and JS drafted the paper. CR-M, JG-G, BL, and JD critically reviewed the paper and approved the final version submitted for publication. All authors contributed to the article and approved the submitted version.

## Conflict of Interest

The authors declare that the research was conducted in the absence of any commercial or financial relationships that could be construed as a potential conflict of interest.
